# Irrigant dynamics of a back-to-back double side-vented needle in root canals with various tapers: a computational fluid dynamics study

**DOI:** 10.1007/s10266-024-00940-2

**Published:** 2024-05-04

**Authors:** Surmayee Singh, Mohammad Zuber, Prathmesh Pravin Verekar, Tejaswini Shetty, N. Srikant, Manuel S. Thomas

**Affiliations:** 1 Department of Conservative Dentistry and Endodontics, Manipal College of Dental Sciences Mangalore, Manipal Academy of Higher Education, Manipal, Karnataka 576104 India; 2 Department of Aeronautical and Automobile Engineering, Manipal Institute of Technology, Manipal, Manipal Academy of Higher Education, Manipal, Karnataka 576104 India; 3 Department of Oral Pathology, Manipal College of Dental Sciences Mangalore, Manipal College of Dental Sciences Mangalore, Manipal Academy of Higher Education, Manipal, Karnataka 576104 India

**Keywords:** Computational fluid dynamics, Irrigation, Needle, Root canal, Syringe

## Abstract

Understanding the apical pressure and irrigant flow patterns in root canals is crucial for safe and effective irrigation. Therefore, this study aimed to assess the flow characteristics of irrigants in root canal models with varying tapers during final irrigation by employing various needle designs, including a back-to-back double-side-vented needle, through computational fluid dynamics. The root canal model was configured as a closed geometrical cone with a simulated apical zone (size 30) and features tapers of 4%, 6%, and 8%. Three needle types—open-ended needle (OEN), single side-vented needle (SSVN), and double side-vented needle (DSVN)—were investigated. The results indicated that for the 4% taper models, the open-ended needle generated the maximum apical pressure, followed by the double side-vented needle and the single side-vented needle. However, in the 6% and 8% tapering root canal models, the double-side-vented needle applied the lowest maximum apical pressure. Consequently, the DSVN can pose a risk for irrigant extrusion in minimally prepared canals due to heightened apical pressure. In wider canals, the DSVN exhibited lower apical pressure. The maximum irrigant replacement was observed with OEN compared to that of the closed-ended group for both flow rates. Additionally, compared with OENs, closed-ended needles exhibited nonuniform and lower shear wall stress.

## Introduction

Along with instrumentation, root canal irrigation is critical for eliminating bacteria, necrotic tissue, organic matter, and inorganic debris from the canal system [1–3]. The most widely used irrigant during endodontic treatment is sodium hypochlorite (NaOCl) [3–5]. NaOCl acts as a solvent for pulp tissue, lubricant, and antimicrobial agent [3, 6]. However, extrusion beyond the apex can include chemical burns, tissue necrosis, and neurological complications such as paraesthesia of the nerves [7, 8].

Despite the danger of apical extrusion due to positive pressure, needle irrigation is still widely accepted by dentists because it is easy to use and cost-effective [4, 5]. There are two types of irrigation needles: (i) open-ended and (ii) closed-ended needles. The open-ended can be flat, bevelled, or notched. The closed-ended needles could be single-side-vented, double-side-vented, or multi-vented needles [9, 10]. Parameters such as flow pattern, velocity, and apical pressure are affected by the tip of the irrigation needle [11, 12]. Limited studies have been performed on a newer double-side-vented needle (Dentsply Sirona irrigation needle), which is a flexible 30-gauge (G) needle with a 4% taper and 27 mm length made of polypropylene body. It also has two side vents that are placed back-to-back at the tip to deliver powerful lateral irrigation [13].

The primary purpose of canal shaping is to provide space for the irrigant delivery system to penetrate the apical third of the root canal for effective irrigation [3, 14]. Lesser tapered canal preparations are currently preferred to greater tapered preparations to aid in the preservation of the critical pericervical dentin [15]. Computational fluid dynamics (CFD) has been shown to be a dependable means for investigating irrigant flow patterns, canal cleaning ability, and the risk of extrusion [9–12].

Therefore, the objective of this study was to determine the flow characteristics of the irrigant in root canal models with varying tapers during final irrigation with various needle designs, including a back-to-back double side-vented 30-G needle, using CFD.

## Materials and methods

**Fig. 1:**
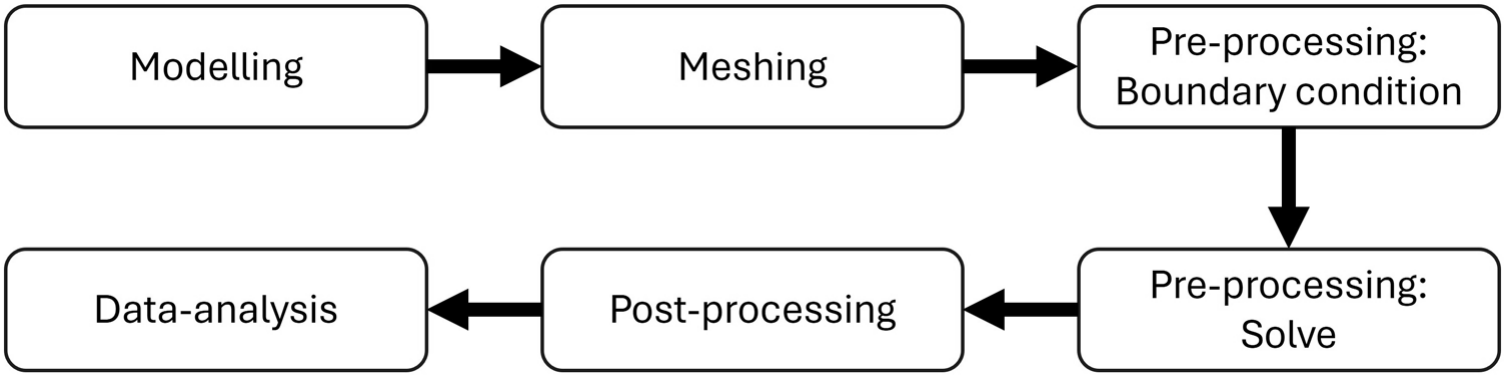
Flow diagram for the steps in computational fluid dynamics study

*Study approval and design:* This computational fluid dynamics (CFD) investigation was initiated following the receipt of ethical approval from the Institutional Research Board (Reference number: *20076*). The steps involved in a CFD study are shown in Fig. 1.

*Design of the CFD root canal model:* For modelling, Ansys® Design Modeller 2022 R1 (ANSYS Inc., Canonsburg, PA, USA) was used to create the needle models. The root canal was designed as an inverted geometrical cone with a cone attached to simulate the apical zone. The root canal length was set at 15 mm, with a 0.3 mm diameter at the most apical point and a 0.9 mm diameter 15 mm from the apex. The taper of the prepared canal varied from 4%, 6%, and 8%. The shape was consistent with the final size obtained after cleaning and shaping with rotary-driven file size no. 30. The size of the minor foramen was set at 0.2 mm, and that of the major foramen was kept at 0.25 mm. The length of the truncated cone was set to 0.5 mm.

*Geometry of the irrigation needles:* Three different irrigation needles were modelled for reference: open-ended (OEN), single side-vented (SSVN), and back-to-back double side-vented needle (DSVN). The details and measurements of the needles used in the study are provided in Table 1. The dimensions of the OEN and SSVN were obtained from a previous study [9]. The dimensions of the DSVN were obtained using a stereomicroscope of 20 × magnification (Stereo Star Zoom-570, Reichert, New York, USA) and ImageJ 1.51j8 software (Wayne Rasband, National Institute of Health, USA).

**Table 1:** Details of the irrigation needles used in the present study

Type of needle	Manufacture details	Diameter (external)	Diameter (internal)	Length of vent	Distance of vent from the tip
Open-ended (OEN)	Navitip, Ultradent, South Jordan, UT	0.320 mm	0.196 mm	–	–
Single side-vented (SSVN)	KerrHawe Irrigation Probe; KerrHawe SA, Bioggio, Switzerland	0.320 mm	0.196 mm	0.96 mm	0.5 mm
Back-to-back double side-vented (DSVN)	Maillefer Irrigation needle, Dentsply Sirona	0.294 mm	0.1 mm	0.513 mm	0.252 mm

*Mesh Construction:* Ansys® Workbench 2022 R1 (ANSYS Inc., Canonsburg, PA, USA) was utilized to generate the mesh. Grid refinement was performed on the domain after constructing the structured mesh. Approximately 0.6 million mesh points were used to simulate the whole model. The maximum skewness was kept below 0.9 to maintain mesh quality.

*Boundary and operating conditions*: No slip boundary conditions were applied to the canal walls or needles. Based on the assumption that root canal walls are rigid, smooth, and impermeable, this experiment was conducted. An inlet velocity of 2.7 m/s and 8.6 m/s, corresponding to irrigation inlet flow rates of 0.083 ml/s and 0.26 ml/s, respectively, were used for the study. A pressure outlet was used for the root canal exit. The irrigant used was a 1% sodium hypochlorite solution with a density of 1.04 g cm^−3^ [9]. This fluid was assumed to completely fill the root canals and was represented as an incompressible Newtonian fluid in the model.

*Variables and outcome processing:* The depth of needle penetration was maintained at (a) 3 mm from the apex, (b) 2 mm from the apex, and (c) 1 mm from the apex. The various outcomes measured were (1) the apical pressure in Kilopascal (KPa), (2) the shear wall stress in Pascal (Pa), (3) the fluid velocity (m/s), and (4) the flow distribution and irrigant penetration. The outcomes were compared for changes in the canal taper, irrigant flow rate, and depth of needle penetration, in addition to the needle design.

*Data analysis:* The flow field values obtained for each of the scenarios were recorded, tabulated, and compared with respect to the apical pressure, wall shear stress, irrigant velocity, and penetration. Since computational fluid dynamics model assessments produce a singular result for each experimental configuration, statistical analysis was not performed [10].

## Results

The results obtained for apical pressure, shear wall stress, velocity vector, and streamline are provided below.

*Apical pressure:* The irrigant pressure applied perpendicular to the apical wall at the apical foramen is known as apical pressure [10]. The apical pressure for each type of needle decreased as the taper increased from 4 to 8%. As the needle approached the apex, there was an increase in apical pressure, with the maximum pressure occurring 1 mm from the apex at both velocities. Additionally, as the velocity increased, the apical pressure increased (Table 2).

**Table 2:** Apical pressure (kPa) in 4, 6, and 8% tapered preparations at distances of 1, 2, and 3 mm from the apex and velocities of 0.083 and 0.26 ml/s for various needles

Taper	Distance from the apex (mm)	Flow rate	Open-ended needle (OEN)	Single side-vented needle (SSVN) and	Double side-vented needle (DSVN)
4%	1	0.083 ml/s	1303.11	111.69	1074.74
		0.26 ml/s	5898.40	527.28	3973.54
	2	0.083 ml/s	170.25	49.13	149.76
		0.26 ml/s	855.88	237.70	609.24
	3	0.083 ml/s	61.26	25.96	42.77
		0.26 ml/s	329.92	128.87	180.69
6%	1	0.083 ml/s	264.85	31.74	27.43
		0.26 ml/s	1343.78	164.11	115.48
	2	0.083 ml/s	44.50	14.59	8.11
		0.26 ml/s	249.86	75.72	35.57
	3	0.083 ml/s	17.48	8.22	3.65
		0.26 ml/s	103.63	42.36	17.21
8%	1	0.083 ml/s	98.44	14.79	6.89
		0.26 ml/s	544.61	79.61	30.29
	2	0.083 ml/s	18.50	6.90	2.37
		0.26 ml/s	110.82	41.82	11.62
	3	0.083 ml/s	8.87	4.20	1.24
		0.26 ml/s	66.39	30.19	7.09

A comparison of the three different types of needles revealed that the maximum apical pressure was achieved by the open-ended needle followed by the double side-vented needle and then the single side-vented needle for the 4% taper models. However, for the 6% and 8% tapering root canal models, the lowest apical pressure was observed for the double-sided-vented needle (Table 2). Elevated apical pressure poses the threat of irrigant extrusion into periapical tissues.

*Shear wall stress:* The magnitude of shear wall stress plays a role in the mechanical removal of debris, tissue remnants, and biofilms. The shear wall stress for all three needle designs decreased with increasing tapering during preparation. The maximum value of shear wall stress was at 1 mm from the apex, and the minimum was at 3 mm from the apex. There was an increase in the shear wall stress with increasing velocity from 0.083 ml/s to 0.26 ml/s (Table 3).

**Table 3:** Maximum wall shear stress and maximum velocity magnitude in 4, 6, and 8% tapered preparations at distances of 1, 2, and 3 mm from the apex and velocities of 0.083 and 0.26 ml/s for various needles

Taper	Distance from the apex (mm)	Flow rate	Maximum wall shear stress (kPa)			Velocity magnitude (m/s)		
			Open-ended needle	Single side-vented needle	Double side-vented needle	Open-ended needle	Single side-vented needle	Double side-vented needle
4%	1	0.083 ml/s	36.2	9.5	1.6	36	14	4.2
		0.26 ml/s	129	30.2	5.9	116	41	12
	2	0.083 ml/s	5.01	3.4	0.6	11	8.3	2.3
		0.26 ml/s	20.9	11.5	2.2	38	25	6.7
	3	0.083 ml/s	2.6	2.5	0.4	6.8	6	1.5
		0.26 ml/s	10	10	1.5	22	20	5.1
6%	1	0.083 ml/s	10.1	3.1	0.7	17	8.5	2.3
		0.26 ml/s	39.4	12.8	2.5	57	25	6.8
	2	0.083 ml/s	2.5	2.3	0.3	6.8	5.7	1.6
		0.26 ml/s	10.4	8.9	1.5	22	19.5	5.1
	3	0.083 ml/s	1.1	1.9	0.3	4.2	4.9	1.5
		0.26 ml/s	6.4	7.3	1.4	13	17	4.9
8%	1	0.083 ml/s	5.2	2.4	0.4	11	6.5	1.6
		0.26 ml/s	22.9	9.9	1.6	38	22	5.1
	2	0.083 ml/s	1.9	1.9	0.3	4.8	5	1.5
		0.26 ml/s	6.4	7.6	1.4	15	17	4.9
	3	0.083 ml/s	1.9	1.9	0.2	4.3	1.7	1.5
		0.26 ml/s	6.4	6.4	1.1	13	14	4.6

**Fig. 2:**
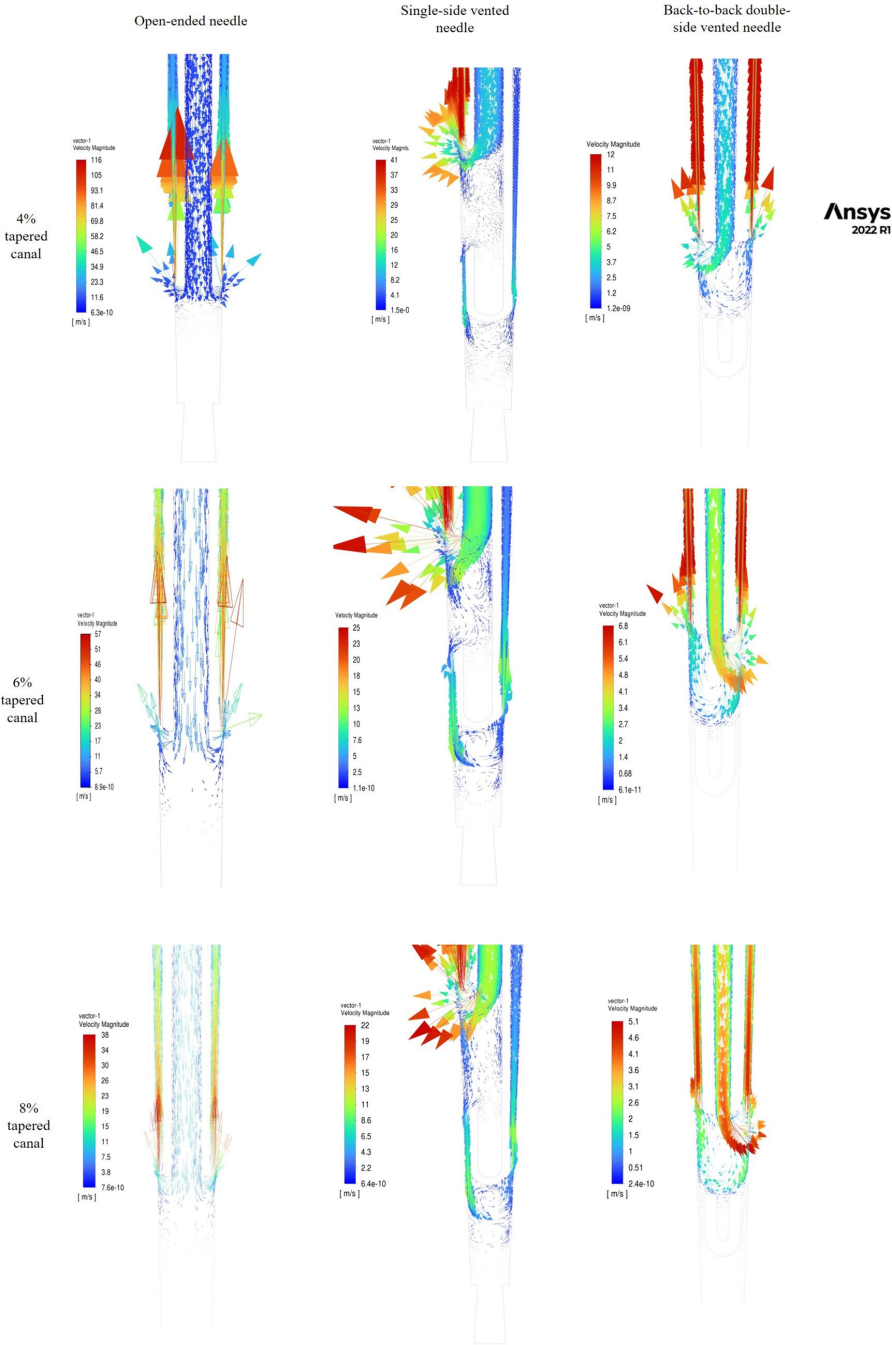
Velocity magnitude of the irrigant at 1 mm from the apex at a flow rate of 0.26 ml/s for the open-ended needle, single side-vented needle, and double side-vented needle in canals with various tapers

A comparison of all three parameters for all three needle designs revealed that the shear stress was greatest for the open-ended needle at a 4% taper of the root canal at 1 mm from the apex when the irrigant velocity was maintained at 0.26 ml/s, which was 129,000 Pa. The minimum shear wall stress was reached for a double-sided ventilated needle at 8% taper height, 3 mm from the apex, and a velocity of 0.083 ml/s (Table 3). Regardless of the needle design, as the needle depth increased, the wall shear stress also increased. The shear stress patterns corresponded to the area of maximum velocity. Thus, for the open-ended needle, wall shear stress was observed apical to the needle outlet. For the closed-ended needles, shear stresses were observed on the canal wall opposite the vent (Fig. 2).

*Velocity:* The inlet velocity of the needle plays a role in determining the flow field of the irrigant and the velocity magnitude as the fluid exits from the needle [11]. With an increase in the inlet velocity of the irrigant from 2.7 m/s to 8.6 m/s, the jet became more intense (Table 3). With the increase in the taper of the preparation of the root canal, the velocity magnitude decreased for all three types of needles (Fig. 2). As the needle moved closer to the apex from 3 to 1 mm, the irrigant velocity increased, indicating improved irrigant replacement. Among the needles tested, the velocity magnitude was greatest for OEN, followed by that for SSVN, and it was least for DSVN. Thus, the stagnation zone was more prominent in the double-side-vented needles.

**Fig. 3:**
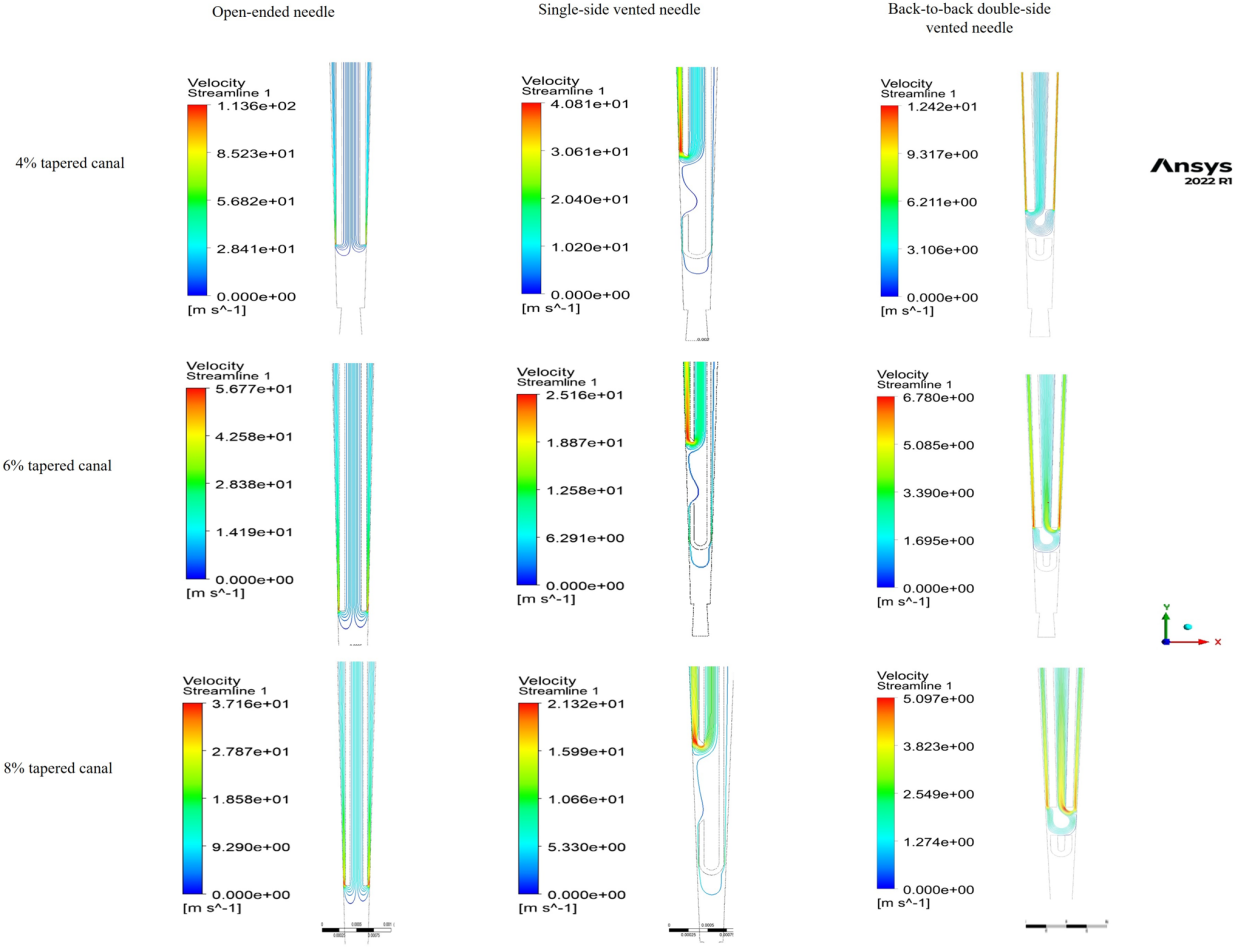
Streamlines of the irrigant at 1 mm from the apex at a flow rate of 0.26 ml/s for the open-ended needle, single side-vented needle, and double side-vented needle in canals with various tapers

*Streamlines:* Streamlines represent the route of particles released downstream from the needle inlet and represent the flow of irrigant apically [11]. Upon comparing all three parameters for all three needle designs, the maximum irrigant penetration was achieved by the open-ended needle, which was limited to less than 1 mm from the needle exit. For the single-side-vented needle, irrigant penetration was limited to less than 0.5 mm apical to the tip of the needle, whereas for the double-side-vented needle, the irrigant barely crossed the tip of the needle (Fig. 3).

## Discussion

There are various methods for studying the fluid flow of irrigants delivered via irrigation needles within the root canal. These include macroscopic methods such as visual observation and spectroscopic methods; the use of contrast media; microscopic methods such as real-time imaging of bioluminescent bacteria; and investigations of the ability of irrigants and devices to remove debris in simulated irregularities [16]. However, the above methods provide incomplete estimates of fluid flow dynamics [17]. This study employed CFD to investigate fluid flow in three distinct needle designs. CFD not only evaluates hydrodynamic performance but also predicts the effectiveness of irrigating needles [10, 16, 17].

As in other studies, in this study, the root canals were modelled as single or straight to avoid complications in the generation of mesh for CFD [9–11]. A standard length of 15 mm for the frustum of the cone was adopted for all the models to simplify the geometry [11]. The apical size was kept corresponding to a #30 file size, as studies have shown this to be the minimum diameter for efficient debridement and disinfection of the canal. This diameter allows the positioning of a 30 G needle nearer to the working length [14, 18]. Due to certain limitations of the model, the apical foramen was considered a rigid and impermeable barrier. Because of such assumptions, the possibility of irrigants being extruded from the apex is excluded [11].

For safe and effective instrumentation, continuous tapering of the root canal with a small diameter at the apex and nominal coronal flaring are required as the fracture resistance of the tooth increases [12, 13]. Newer NiTi files were introduced with a continuous taper (4%) for conservative preparation of the root canal and preservation of the pericervical dentin [15, 19]. In the literature, there are limited CFD studies performed on root canal models with 4% taper canal preparation [20]. Therefore, in this study, different root canal preparations, such as 4%, 6%, and 8% taper canal preparations, were compared.

The different needle designs used in the study were open-ended, single-side-vented, and double-side-vented. For standardization in this study, all three different needle designs used were 30G [10]. The dimensions of the first two needles were reproduced from the study by Boutsioukis et al. [21]. The last needle studied was the Maillefer Dentsply 30G plastic needle with 2 lateral openings [22]. This study is one of the first to investigate the flow dynamics of root canal irrigant using these newly introduced irrigating needles. In the present study, two irrigant flow rates, 0.083 ml/sec and 0.26 ml/sec, were compared; these are clinically realistic flow rates that help in evaluating the effect of an increase in velocity on different fluid dynamics parameters, such as apical pressure, shear wall stress and irrigant flow pattern [11, 23].

A larger volume of fluid penetrates the apical anatomy when an open-ended needle is used, which leads to an increase in kinetic energy and, in turn, an increase in apical pressure. This was in accordance with the findings of Shen et al. [10] and Boutsioukis and his colleagues [11, 24]. Therefore, the utilization of OEN increases the likelihood of irrigant extrusion, especially in high-flow-rate cases [11]. The less tapered canals had greater apical pressure, which may be related to the reduced room at the needle's tip for reverse flow in the direction of canal opening [20]. Additionally, the apical pressure was directly proportional to the depth of insertion of the needle. This finding is consistent with the findings of a previous study that showed greater apical pressure when the needle was positioned closer to the working length [24].

When the DSVN was 1 mm shy of the working length, even at a lower flow rate, the apical pressure was 1074.7 kPa, while the pressure on the SSVN was 111.7 kPa. The probable reason for this marked apical pressure could be the 4% taper of the needle, as opposed to the parallel nature of the other needles tested [13]. This taper provides less space for the backflow of the irrigants toward the orifice, leading to increased apical pressure. The results of this study for apical pressure in a 6% tapered root canal were similar to those of Shen et al., in which the pressure in the closed-ended needle was 2.5–3 times lower than that in the open-ended needle [10]. However, the apical pressure observed in this study surpassed that reported in prior research [10, 24]. This is probably because of the variations in the experimental settings and the CFD model. Overall, in all the situations studied here, the SSVN and DSVN were preferable to the OEN, except in minimally tapered preparations. Similar to the open-ended needle, in both side-vented needles tested, the apical pressure was greater when the flow rate increased, the canal taper decreased, and the needle penetrated deeper. Hence, caution is advised in these scenarios to reduce the risk of irrigant extrusion through the apical foramen [21].

In the present study, the shear stress generated by the OEN was the highest, followed by that induced by the SSVN and then that induced by the DSVN. In the SSVN and DSVN, the stress was mainly concentrated on the walls facing the needle outlet. These observations are similar to those of previous studies [9, 18]. With the increase in needle penetration depth and velocity in all three types of needles, there was an increase in shear stress as well as in the shear wall stress region, which was in accordance with the findings of previous studies [24]. Although the wall shear stress generated by the DSVN was drastically low, the area of stress concentration observed on the walls opposite to the openings of the double-side vented needle was greater. This could lead to a larger area of debris and biofilm detachment, thereby enhancing cleaning [21]. Rotation of these needles while irrigating could also prove beneficial for more effective canal debridement [25, 26].

The magnitude of the irrigant velocity within the root canal is a crucial parameter when assessing flow parameters. It allows the evaluation of the irrigant replacement and reverse flow of the irrigant coronally [2, 11]. Efficient canal cleaning relies on favourable irrigant flow to effectively carry debris from the canals out of the orifice [9]. The irrigant replacement and reverse flow were better with open-ended needles than with closed-ended needles, especially when the flow rate was higher and canals were less tapered. However, from a clinical perspective, the risk of irrigant replacement far outweighs that of irrigant replacement [24]. Hence, when employing an open-ended needle under high irrigant flow rates in minimally tapered canals, operators must exercise caution.

The results of this study also demonstrated that none of the needles tested with various parameters could effectively deliver the irrigant up to the apex. The OEN demonstrated greater irrigant penetration than did the side-vented needles. This result was supported by previous studies in which side-vented needles reported limited irrigant exchange [9, 11]. The single-sided vented needle showed better apical penetration of the irrigant than did the double-sided vented needle. For the DSVN, the irrigant barely crossed the tip of the needle. The inability of all the needles examined in this study to effectively replace irrigants even when the tip is 1 mm from the apical construction contradicts the findings of previous studies [2, 20]. The conflicting findings could be attributed to the varied experimental settings and variations in the numerical models used [2]. As mentioned above, all the irrigating needles demonstrated a stagnant dead water zone apical to the tip [27]. Therefore, it is imperative to agitate irrigants or even slowly move the needle up and down within canals to effectively clean the root canal to its terminus and to improve fluid replacement dynamics. [28, 29].

The closed-ended double-side-vented needle tested in this study differed from that used in previous studies [2, 9, 21] in that the openings in this needle are positioned back-to-back rather than at the top and bottom. In the latter double-vented needle, the flow between the two openings is not equally distributed. The fluid passes more through the vent closer to the tip of the needle [21]. However, in the back-to-back side vented needle, the flow from both vents is equally distributed. The overall size of the opening could also account for the lower wall shear stress and irrigant velocity. The advantage of the back-to-back double-side-vented needle tested in the current study is that it has a flexible body made of polypropylene that allows the needle to access the apical regions without resistance or damage to the dentinal walls even in curved canals [22, 30]. Therefore, further research is required with regard to the irrigant flow and efficiency in curved canals using these syringe needles.

The limitations of the study include the design of the root canal model, which was considered to be closed and straight, with impermeable and rigid walls. Surface irregularities were also not incorporated into the model design. The parameters of irrigation dynamics can easily be affected by such changes. Additionally, it is difficult to maintain constant inlet flow velocities in clinical scenarios. Therefore, the quantitative data obtained from these studies cannot be directly applied in clinical situations.

## Conclusion

Considering the limitations of the current study, the following conclusions can be drawn:

- The canal taper, needle type, needle insertion depth, and irrigant flow rate were found to affect the pressure at the apical foramen, the shear stress on the canal wall, and the extent of irrigant replacement.
- The apical pressure was lower in the single-side-vented needle group than in the open-end needle group. Therefore, its use is safe, but its efficiency in canal debridement could be questionable.
- The back-to-back double-side-vented tapered needle should be used with caution in minimally prepared canals, as there could be a risk of irrigant extrusion due to exaggerated apical pressure.
- All the needles tested for various parameters failed to deliver irrigant to the apex.

## Data Availability

The data regarding this research article will be provided upon formal request from the corresponding author.

## References

[1] Gulabivala K, Patel B, Evans G, Ng YL. Effects of mechanical and chemical procedures on root canal surfaces. Endod Topics. 2005;10:103–22.

[2] Haapasalo M, Shen Y, Wang Z, Gao Y. Irrigation in endodontics. Br Dent J. 2014;216:299–303. 10.1038/sj.bdj.2014.204. 24651335 10.1038/sj.bdj.2014.204

[3] Boutsioukis C, Arias-Moliz MT. Present status and future directions - irrigants and irrigation methods. Int Endod J. 2022;55:588–612. 10.1111/iej.13739. 35338652 10.1111/iej.13739 PMC9321999

[4] Dutner J, Mines P, Anderson A. Irrigation trends among American association of endodontists members: a web-based survey. J Endod. 2012;38:37–40. 10.1016/j.joen.2011.08.013. 22152617 10.1016/j.joen.2011.08.013

[5] Gopikrishna V, Pare S, Pradeep Kumar A, Lakshmi NL. Irrigation protocol among endodontic faculty and post-graduate students in dental colleges of India: a survey. J Conserv Dent. 2013;16:394–8. 10.4103/0972-0707.117486. 24082565 10.4103/0972-0707.117486 PMC3778618

[6] Zehnder M. Root canal irrigants. J Endod. 2006;32:389–98. 10.1016/j.joen.2005.09.014. 16631834 10.1016/j.joen.2005.09.014

[7] Guivarc’h M, Ordioni U, Ahmed HM, Cohen S, Catherine JH, Bukiet F. Sodium hypochlorite accident: a systematic review. J Endod. 2017;43:16–24. 10.1016/j.joen.2016.09.023. 27986099 10.1016/j.joen.2016.09.023

[8] Reda R, Zanza A, Bhandi S, Biase A, Testarelli L, Miccoli G. Surgical-anatomical evaluation of mandibular premolars by CBCT among the Italian population. Dent Med Probl. 2022;59:209–16. 10.17219/dmp/143546. 35766896 10.17219/dmp/143546

[9] Boutsioukis C, Verhaagen B, Versluis M, Kastrinakis E, Wesselink PR, van der Sluis LW. Evaluation of irrigant flow in the root canal using different needle types by an unsteady computational fluid dynamics model. J Endod. 2010;36:875–9. 10.1016/j.joen.2009.12.026. 20416437 10.1016/j.joen.2009.12.026

[10] Shen Y, Gao Y, Qian W, et al. Three-dimensional numeric simulation of root canal irrigant flow with different irrigation needles. J Endod. 2010;36:884–9. 10.1016/j.joen.2009.12.010. 20416439 10.1016/j.joen.2009.12.010

[11] Boutsioukis C, Lambrianidis T, Kastrinakis E. Irrigant flow within a prepared root canal using various flow rates: a computational fluid dynamics study. Int Endod J. 2009;42:144–55. 10.1111/j.1365-2591.2008.01503.x. 19134043 10.1111/j.1365-2591.2008.01503.x

[12] Çiftçioğlu E, Yücel Ö, Işık V, Keleş A, Kayahan MB. Irrigant flow characteristics in the root canal with internal root resorption: a computational fluid dynamics evaluation. Odontology. 2022;110:769–76. 10.1007/s10266-022-00698-5. 35218447 10.1007/s10266-022-00698-5

[13] Dentsply sirona irrigation needle. Available at https://www.dentsplysirona.com/en-in/categories/endodontics/irrigation-needle.html. Accessed on September 10, 2023

[14] Khademi A, Yazdizadeh M, Feizianfard M. Determination of the minimum instrumentation size for penetration of irrigants to the apical third of root canal systems. J Endod. 2006;32:417–20. 10.1016/j.joen.2005.11.008. 16631839 10.1016/j.joen.2005.11.008

[15] Celikten B, Koohnavard M, Oncu A, Sevimay FS, Orhan AI, Orhan K. A new perspective on minimally invasive endodontics: a systematic review. Biotechnol Biotechnol Equip. 2021;35:1758–67. 10.1080/13102818.2021.2014966.

[16] Boutsioukis C, Arias-Moliz MT, Chávez de Paz LE. A critical analysis of research methods and experimental models to study irrigants and irrigation systems. Int Endod J. 2022;55:295–329. 10.1111/iej.13710. 35171506 10.1111/iej.13710 PMC9314845

[17] Shen C, Gao B, Lv K, Yao H. Computational fluid dynamics in root canal irrigation. Int J Numer Method Biomed Eng. 2023. 10.1002/cnm.3738. 37310003 10.1002/cnm.3738

[18] Boutsioukis C, Gogos C, Verhaagen B, Versluis M, Kastrinakis E, Van der Sluis LW. The effect of apical preparation size on irrigant flow in root canals evaluated using an unsteady computational fluid dynamics model. Int Endod J. 2010;43:874–81. 10.1111/j.1365-2591.2010.01761.x. 20618879 10.1111/j.1365-2591.2010.01761.x

[19] Nassar S, Shetty HK, Nair PMS, Gowri S, Jayaprakash K. Comparative evaluation of fracture resistance of endodontically treated bicuspids instrumented with hand files, trunatomy, protaper next, protaper gold, and waveone - an in vitro study. J Pharm Bioallied Sci. 2022;14:S600–4. 10.4103/jpbs.jpbs_739_21. 36110709 10.4103/jpbs.jpbs_739_21 PMC9469274

[20] Boutsioukis C, Gogos C, Verhaagen B, Versluis M, Kastrinakis E, Van der Sluis LW. The effect of root canal taper on the irrigant flow: evaluation using an unsteady computational fluid dynamics model. Int Endod J. 2010;43:909–16. 10.1111/j.1365-2591.2010.01767.x. 20618877 10.1111/j.1365-2591.2010.01767.x

[21] Boutsioukis C, Gutierrez NP. Syringe irrigation in minimally shaped root canals using 3 endodontic Needles: a computational fluid dynamics study. J Endod. 2021;47:1487–95. 10.1016/j.joen.2021.06.001. 34118256 10.1016/j.joen.2021.06.001

[22] Diemer F, Diemer M, Beaugendre A, Blasco-Baqué V, Mallet JP. Irrigation in endodontics- new needle for better results. Roots Int. 2019;2019:22–4.

[23] Boutsioukis C, Kastrinakis E, Lambrianidis T, Verhaagen B, Versluis M, van der Sluis LW. Formation and removal of apical vapor lock during syringe irrigation: a combined experimental and computational fluid dynamics approach. Int Endod J. 2014;47:191–201. 10.1111/iej.12133. 23711027 10.1111/iej.12133

[24] Boutsioukis C, Lambrianidis T, Verhaagen B, et al. The effect of needle-insertion depth on the irrigant flow in the root canal: evaluation using an unsteady computational fluid dynamics model. J Endod. 2010;36:1664–8. 10.1016/j.joen.2010.06.02. 20850673 10.1016/j.joen.2010.06.023

[25] Wang R, Shen Y, Ma J, et al. Evaluation of the effect of needle position on irrigant flow in the C-shaped root Canal using a computational fluid dynamics model. J Endod. 2015;41:931–6. 10.1016/j.joen.2015.02.002. 25791077 10.1016/j.joen.2015.02.002

[26] Patrício M, Santos JM, Oliveira P, Patrício F. Computational fluid dynamics in root canal procedures. Proceedings of the 11th International Conference on Computational and Mathematical Methods in Science and Engineering, CMMSE2011; 2011 Jun 26–30; Benidorm, Spain.

[27] Yu M, Li Y, Zhao M, Huang Z, Zhou N, Jin H. Computational fluid dynamics investigation on the irrigation of a real root canal with a side-vented needle. BMC Oral Health. 2024;24:321. 10.1186/s12903-024-03966-8. 38461300 10.1186/s12903-024-03966-8 PMC10924978

[28] Zou X, Zheng X, Liang Y, et al. Expert consensus on irrigation and intracanal medication in root canal therapy. Int J Oral Sci. 2024;16:23. 10.1038/s41368-024-00280-5. 38429299 10.1038/s41368-024-00280-5 PMC10907616

[29] Hu S, Duan L, Wan Q, Wang J. Evaluation of needle movement effect on root canal irrigation using a computational fluid dynamics model. Biomed Eng Online. 2019;18:52. 10.1186/s12938-019-0679-5. 31060550 10.1186/s12938-019-0679-5 PMC6501388

[30] Provoost C, Rocca GT, Thibault A, Machtou P, Bouilllaguet S. Influence of needle design and irrigant flow rate on the removal of enterococcus faecalis biofilms in vitro. Dent J (Basel). 2022;10:59. 10.3390/dj10040059. 35448053 10.3390/dj10040059 PMC9030241

